# Evaluation of Atlas‐based auto‐segmentation of liver in MR images for Yttrium‐90 selective internal radiation therapy

**DOI:** 10.1002/acm2.13979

**Published:** 2023-04-17

**Authors:** Jun Li, Rani Anne

**Affiliations:** ^1^ Department of Radiation Oncology Thomas Jefferson University Philadelphia Pennsylvania USA

**Keywords:** atlas segment, auto‐segmentation, liver delineation, MR image, resin Yttrium‐90

## Abstract

**Purpose:**

The aim was to explore the feasibility of applying an atlas‐based auto‐segmentation tool, MIM Atlas Segment, for liver delineation in MR images in Y‐90 selective internal radiation therapy (SIRT).

**Materials and methods:**

MR images of 41 liver patients treated with resin Y‐90 SIRT were included: 20 patients’ images were used to create an atlas, and the other 21 patients’ images were used for testing. Auto‐segmentation of liver in the MR images was performed with MIM Atlas Segment, and various settings for the auto‐segmentation (i.e., with and without normalized deformable registration, single atlas‐match and multi‐atlas match, and multi‐atlas match using different finalization methods) were tested. Auto‐segmented liver contours were compared with physician manually‐delineated contours, using Dice similarity coefficient (DSC) and mean distance to agreement (MDA). Ratio of volume (RV) and ratio of activity (RA) were calculated to further evaluate the auto‐segmentation results.

**Results:**

Auto‐segmentations with normalized deformable registration generated better contours than those without normalized deformable registration. With normalized deformable registration, 3‐atlas match using Majority Vote (MV) method generated better results than single‐atlas match and 3‐atlas match using STAPLE method, and generated similar results as 5‐atlas match using MV method or STAPLE method. The average DSC, MDA, and RV of the contours generated with normalized deformable registration are 0.80‐0.83, 0.60‐0.67, and 0.91‐1.00 cm, respectively. The average RA are 1.00‐1.01, which indicate that the activities calculated using the auto‐segmented liver contours are close to the accurate activities.

**Conclusion:**

The atlas‐based auto‐segmentation can be applied to generate initial liver contours in MR images for resin Y‐90 SIRT, which can be used for activity calculations after physicians review.

## 1 INTRODUCTION

Yttrium‐90 (Y‐90) selective internal radiation therapy (SIRT) is a radioembolization procedure using Y‐90 microspheres to treat non‐resectable primary and metastatic liver cancers.^1^ In a resin based Y‐90 SIRT procedure where body‐surface‐area method is used for dosimetry, liver volume size and tumor volume size are needed in the activity calculation.^2^ To obtain the volumes, physicians need to delineate contours in CT or MR images. It is desired that an auto‐segmentation tool can be applied for liver delineation in SIRT procedures.

Auto‐segmentation methods have been studied for target and organ delineations in radiation therapy, for example, in prostate, head and neck, pelvis, and brain treatments, etc.^3^, ^4^, ^5^, ^6^, ^7^, ^8^, ^9^ Studies of liver auto‐segmentation have been reported,^10^, ^11^, ^12^, ^13^, ^14^ most of which were based on researcher‐developed methods.

MIM Maestro (MIM Software Inc., Cleveland, OH, USA) is a commercial software popularly used in radiation therapy. MIM software with a regular license provides an atlas‐based auto‐segmentation tool, Atlas Segment. A study of using Atlas Segment for liver delineation in CT images was conducted recently.^15^ In SIRT, MR images are preferred as they provide better soft tissue contrast than CT images. The aim of the study was to explore the feasibility of applying MIM Atlas Segment in MR images, for potential applications in liver delineation for resin Y‐90 SIRT. To the authors’ knowledge, applications of MIM Atlas Segment for liver delineation in MR images have not been reported. The study was also aimed to optimize the auto‐segmentation by testing various settings. Knowledge obtained in the study will be helpful to the application of the auto‐segmentation in SIRT and other procedures.

## 2 METHODS

Auto‐segmentation of liver in MR images was performed with MIM Maestro Atlas Segment (version 6.67). MR images of 41 patients were included, which were randomly selected from the patients who were treated with resin Y‐90 in our institution in recent years. Table 1 lists patient characteristics. Among them, MR images of 20 patients were used to create an atlas. MR images of the other 21 patients were used for testing.

**TABLE 1:** Characteristics of the patients (*N* = 41)

	Range (average ± std)
Age	34‐84 (66 ± 12)
Liver volume size (cm^3^)	999‐3070 (1586 ± 455)

No image processing was performed on the images. During the creation of the atlas, based on patients’ sizes, liver sizes and liver shapes, we selected one patient's images as the representative subject, that is, atlas template. The rest 19 patients’ images (i.e., atlas subjects) were registered to the template with a rigid algorithm, and similarity indices were determined by the software. During an auto‐segmentation, the images of a test case were registered to the atlas template and a similarity index was determined. The software compared the similarity index with those of the atlas subjects and selected an atlas subject which best matched the test case. Then the software performed image registration between the test case and the atlas subject, and deformed the contour of the atlas subject to the test case.

In the MIM Atlas Segment, there are a few setting options for a user to choose for an auto‐segmentation, for example, selecting Normalization (i.e., use Normalized Deformable Registration) or not (the default setting), and entering a number in Number of Atlas Matches or not (the default). If Normalization is selected, a bi‐linear model will be fit to best normalize the intensities of one image to match those of the other during the deformable registration. If a number (larger than 1), for example, “3” is entered in the Number of Atlas Matches, an auto‐segmentation with 3‐atlas match will be performed. The software will search in the atlas to find 3 atlas subjects which have the best match of similarity index with the test case. Then the software will perform image registrations, deform the contours of the 3 atlas subjects to the test case, and use the Finalize Method to generate the final contour. The default Finalize Method is Majority Vote (MV). Another Finalize Method, Simultaneous Truth and Performance Level Estimation (STAPLE), can be selected instead of MV. The MV method checks each voxel in the target. If the voxel belongs to more than half of the contours, the voxel will be included in the final contour. The STAPLE method computes a probabilistic estimate of the true representation of the contour combination, which is formed by estimating an optimal combination of the contours, weighting each contour depending on the estimated performance level. When the estimate is within a tolerance, the final contour is created.

In the study, several settings were tested. Firstly, auto‐segmentations were conducted without normalized deformable registration: (1A) no Normalization (the default setting, labeled as “NoNorm” in the study); (1B) no Normalization, 3 atlas match, and MV method (labeled as “NoNorm_Match3_MV”); (1C) no Normalization, 3 atlas match, and STAPLE method (labeled as “NoNorm_Match3‐STAPLE”); (1D) no Normalization, 5 atlas match, and MV method (labeled as “NoNorm_Match5_MV”); and (1E) no Normalization, 5 atlas match, and STAPLE method (labeled as “NoNorm_Match5‐STAPLE”).

Auto‐segmentations were then conducted with normalized deformable registration: (2A) Normalization (“Norm”); (2B) Normalization, 3 atlas match, and MV method (“Norm_Match3_MV”); (2C) Normalization, 3 atlas match, and STAPLE method (“Norm_Match3‐STAPLE”); (2D) Normalization, 5 atlas match, and MV method (“Norm_Match5_MV”); and (2E) Normalization, 5 atlas match, and STAPLE method (labeled as “Norm_Match5‐STAPLE”).

The auto‐segmented liver contours were compared with the manually‐delineated contours using Dice similarity coefficient (DSC) and mean distance to agreement (MDA). The DSC quantifies the overlap between the two contours: one represents a perfect overlap and 0 represents no overlap. MDA represents the average distance between the two contours: the smaller the MDA, the better the contour agreement is.

Based on the results of DSC and MDA, further evaluations were performed on the auto‐segmented contours which had better agreement with the manually‐delineated contours. The liver volumes obtained in the auto‐segmentations were compared to the liver volumes obtained from the manual delineations by ratio of volume (RV). Ratio of activity (RA), that is, ratio of the activity calculated using the auto‐segmented liver volume to the activity calculated using the manually‐delineated liver volume, which was the standard, was calculated and was used to evaluate activity deviations from the accurate values. One represents no deviations.

Contouring time was not compared in this retrospective study because the time spent on the manual‐delineation was not recorded in the SIRT procedures. The study was focused on evaluating the accuracy of the auto‐segmentation.

In the comparisons, Wilcoxon signed rank test was conducted to test difference significance and a significance level of 0.05 was applied.

## 3 RESULTS

**FIGURE 1:**
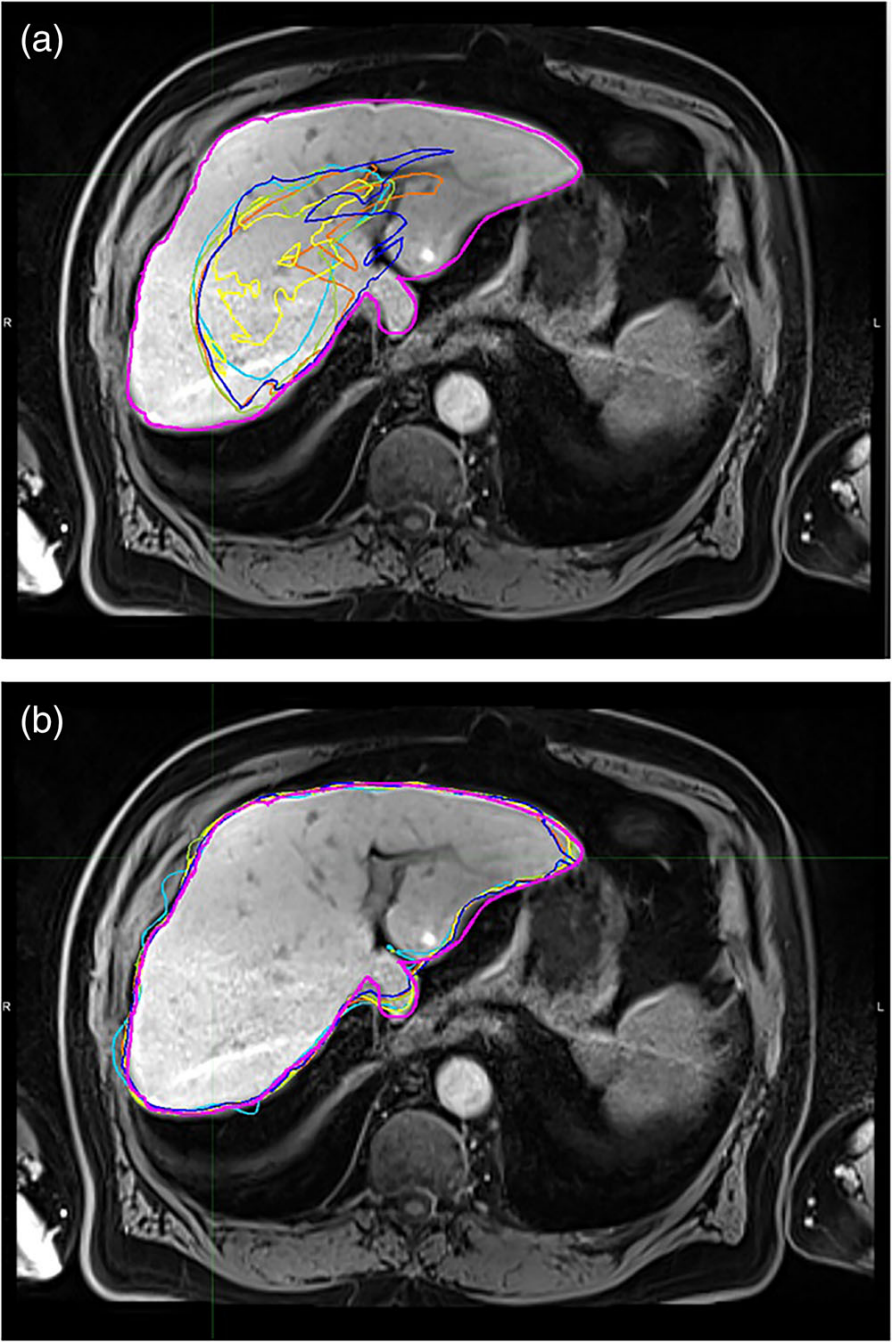
Auto‐segmentation results in one case. (a) Contours generated in the auto‐segmentations without normalized deformable registration: NoNorm (light blue), NoNorm_Match3_MV (red‐orange), NoNorm_Match3_Staple (light green), NoNorm_Match5_MV (yellow), and NoNorm_Match5_Staple (dark blue). (b) Contours generated in the auto‐segmentations with normalized deformable registration: Norm (light blue), Norm_Match3_MV (red‐orange), Norm_Match3_Staple (light green), Norm_Match5_MV (yellow), and Norm_Match5_Staple (dark blue). Manually‐delineated contours are in magenta.

Figure 1 shows auto‐segmented contours in one case. Figure 1a shows contours generated in the auto‐segmentations without normalized deformable registration. Figure 1b shows contours generated in the auto‐segmentations with normalized deformable registration. Manually‐delineated contours are shown as the references. With normalized deformable registration, the auto‐segmentation performances were improved remarkably: the auto‐segmented contours are very similar to the manually‐delineated contour.

**FIGURE 2:**
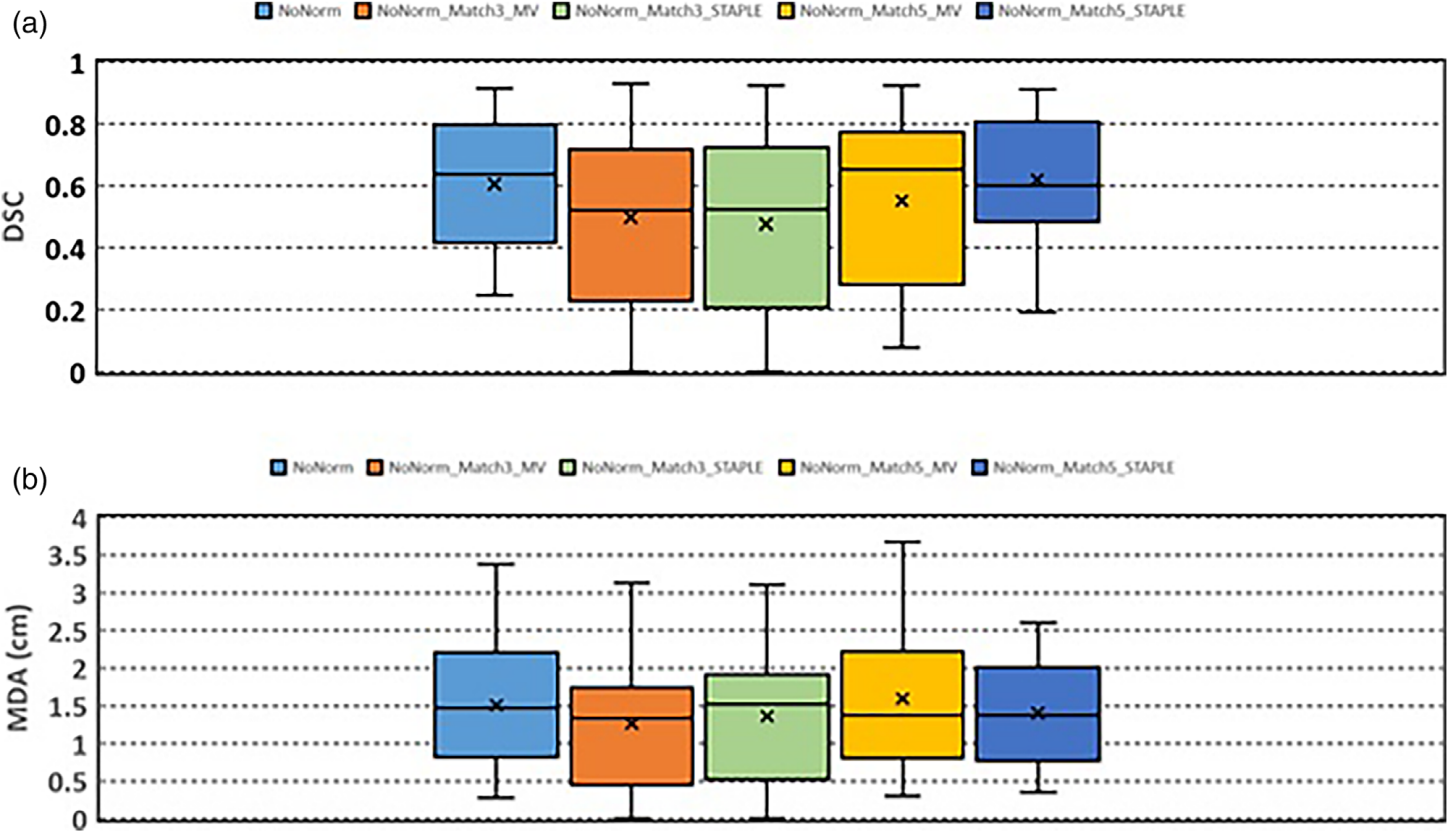
(a) Dice similarity coefficient and (b) mean distance agreement between auto‐segmented liver contours and manually‐delineated liver contours. Normalized deformable registration was not applied in the auto‐segmentation.

Figure 2 and Table 2 show the results of DSC and MDA of the contours generated without normalized deformable registration. DSC are 0.61 ± 0.21 (NoNorm), 0.61 ± 0.23 (NoNorm_Match3_MV), 0.58 ± 0.23 (NoNorm_Match3_STAPLE), 0.55 ± 0.28 (NoNorm_Match5_MV), and 0.62 ± 0.21 (NoNorm_Match5_STAPLE), respectively. MDA are 1.51 ± 0.86 cm (NoNorm), 1.43 ± 0.79 cm (NoNorm_Match3_MV), 1.53 ± 0.86 cm (NoNorm_Match3_STAPLE), 1.59 ± 0.93 cm (NoNorm_Match5_MV), and 1.40 ± 0.69 cm (NoNorm_Match5_STAPLE), respectively. There are no significant differences in DSC and MDA, between single atlas match (NoNorm) and multi‐atlas match, between NoNorm_Match3_MV and other multi‐atlas match, and between NoNorm_Match5_MV and NoNorm_Match5_STAPLE.

**TABLE 2:** DSC and MDA of the auto‐segmented contours generated without normalized deformable registration (*N* = 21)

	NoNorm	NoNorm_Match3_MV	NoNorm_Match3_STAPLE	NoNorm_Match5_MV	NoNorm_Match5_STAPLE
DSC					
mean ± stdev	0.61 ± 0.21	0.61 ± 0.23	0.58 ± 0.23	0.55 ± 0.28	0.62 ± 0.21
median	0.64	0.65	0.59	0.65	0.60
*p*‐value	reference	0.40	0.33	0.33	0.29
		reference	0.06	0.14	0.33
				reference	0.07
MDA (cm)					
mean ± stdev	1.51 ± 0.86	1.43 ± 0.79	1.53 ± 0.86	1.59 ± 0.93	1.40 ± 0.69
median	1.47	1.35	1.65	1.38	1.37
*p*‐value	reference	0.39	0.41	0.41	0.27
		reference	0.10	0.23	0.46
				reference	0.20

**FIGURE 3:**
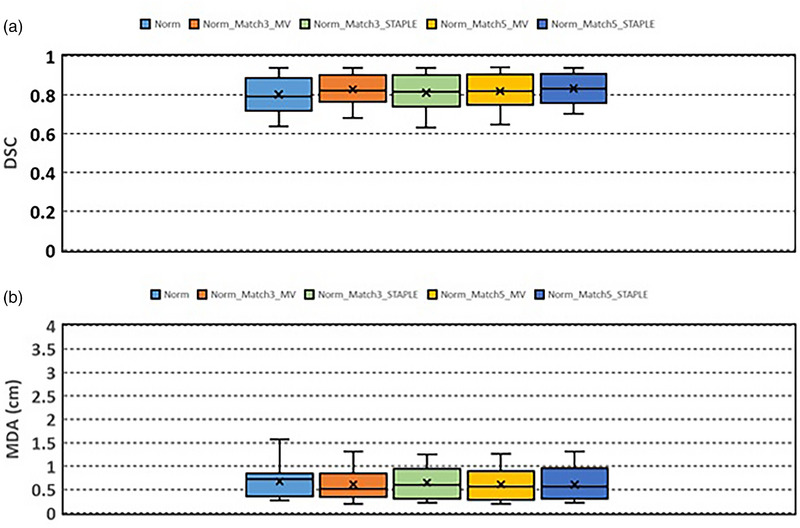
(a) Dice similarity coefficient and (b) mean distance agreement between auto‐segmented liver contours and manually‐delineated liver contours. Normalized deformable registration was applied in the auto‐segmentation.

Figure 3 and Table 3 show the results of DSC and MDA of the contours generated with normalized deformable registration. Compared to the contours generated without normalized deformable registration, DSC and MDA of the contours generated with normalized deformable registration are significantly increased and decreased, respectively. The auto‐segmented contours were significantly improved. DSC are 0.80 ± 0.10 (Norm), 0.83 ± 0.08 (Norm_Match3_MV), 0.81 ± 0.09 (Norm_Match3_STAPLE), 0.82 ± 0.09 (Norm_Match5_MV), and 0.83 ± 0.08 (Norm_Match5_STAPLE), respectively. MDA are 0.67 ± 0.36 cm (Norm), 0.61 ± 0.32 cm (Norm_Match3_MV), 0.65 ± 0.33 cm (Norm_Match3_STAPLE), 0.61 ± 0.33 cm (Norm_Match5_MV), and 0.60 ± 0.34 cm (Norm_Match5_STAPLE), respectively. Compared to the single‐atlas match (Norm), multi‐atlas match except Norm_Match3_STAPLE, generated better contours. There are no significant differences in DSC and MDA between Norm_Match3_MV, Norm_Match5_MV, and Norm_Match5_STAPLE. There are significant differences between Norm_Match3_MV and Norm_Match3_STAPLE, but there are no significant differences between Norm_Match5_MV and Norm_Match5_STAPLE.

**TABLE 3:** DSC and MDA of the auto‐segmented contours generated with normalized deformable registration (*N* = 21)

	Norm	Norm_Match3_MV	Norm_Match3_STAPLE	Norm_Match5_MV	Norm_Match5_STAPLE
DSC					
mean ± stdev	0.80 ± 0.10	0.83 ± 0.08	0.81 ± 0.09	0.82 ± 0.09	0.83 ± 0.08
median	0.79	0.82	0.82	0.82	0.83
*p*‐value	reference	0.02	0.08	0.02	0.01
		reference	0.03	0.42	0.18
				reference	0.08
MDA (cm)					
mean ± stdev	0.67 ± 0.36	0.61 ± 0.32	0.65 ± 0.33	0.61 ± 0.33	0.60 ± 0.34
median	0.71	0.51	0.59	0.55	0.56
*p*‐value	reference	0.03	0.10	0.01	0.03
		reference	0.04	0.24	0.15
				reference	0.48

**FIGURE 4:**
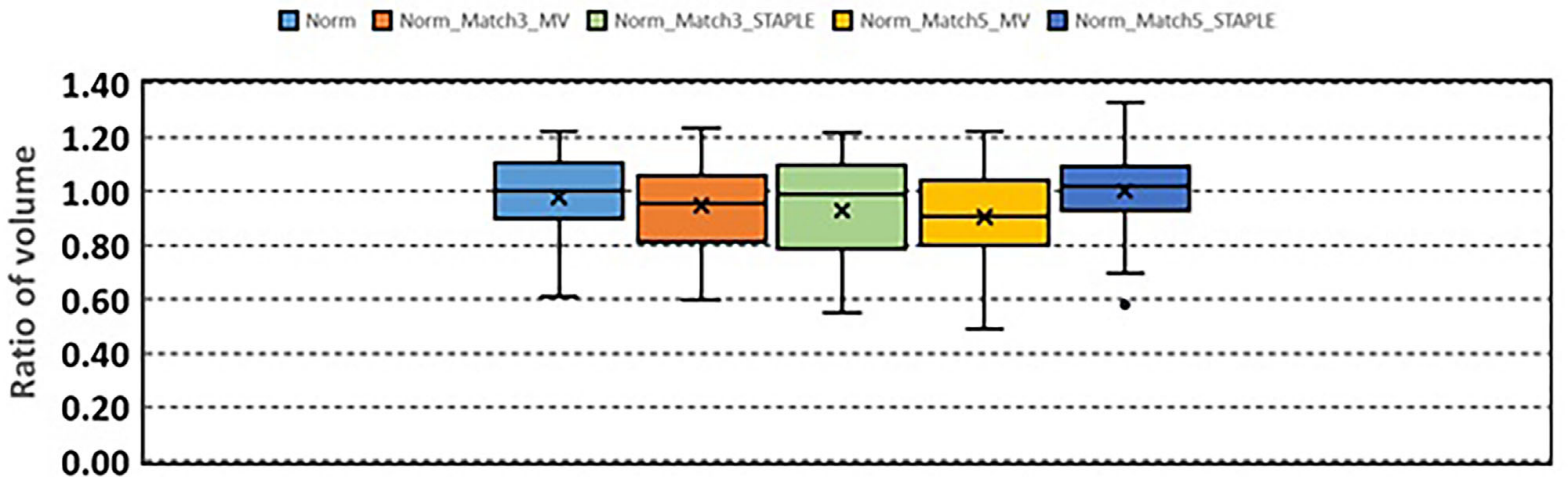
Ratio of the auto‐segmented liver volume which was obtained in the auto‐segmentation with normalized deformable registration, to the manually‐delineated liver volume.

**FIGURE 5:**
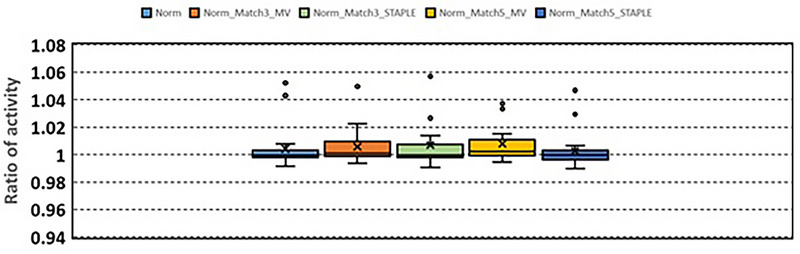
Ratio of the activity calculated using the auto‐segmented liver volume which was obtained in the auto‐segmentation with normalized deformable registration, to the activity calculated using manually‐delineated liver volume.

Compared to the contours generated without using normalized deformable registration, the contours generated with normalized deformable registration had better agreement with the manually‐delineated contours. These contours were further evaluated using RV and RA. Figure 4 shows RV. RV are 0.98 ± 0.16 (Norm), 0.96 ± 0.16 (Norm_Match3_MV), 0.94 ± 0.19 (Norm_Match3_STAPLE), 0.91 ± 0.17 (Norm_Match5_MV), and 1.00 ± 0.18 (Norm_Match5_STAPLE), respectively. Figure 5 shows RA. RA are 1.00 ± 0.02 (Norm), 1.01 ± 0.01 (Norm_Match3_MV), 1.01 ± 0.02 (Norm_Match3_STAPLE), 1.01 ± 0.01 (Norm_Match5_MV), and 1.00 ± 0.01 (Norm_Match5_STAPLE), respectively.

## 4 DISCUSSION

Without using normalized deformable registration, that is, with the default setting, overall the results of the auto‐segmentations are poor (average DSC: 0.55‐0.62; average MDA: 1.40‐1.59 cm). When normalized deformable registration was used, the auto‐segmentations were improved remarkably (average DSC: 0.80‐0.83; average MDA: 0.60‐0.67 cm). The MR images used in the study were contrast‐enhanced images which had been used in the SIRT procedures. The enhanced image contrast might play an important role in the normalized deformable registration.

With normalized deformable registration, the auto‐segmentations with 3‐atlas match using MV method generated better results than those with 3‐atlas match using STAPLE method (*p*‐value: 0.03). The auto‐segmentations with 5‐atlas match using MV method however did not show significant differences from the auto‐segmentations with 5‐atlas match using STAPLE method (*p*‐value: 0.08). The results showed that when the number of atlas match was small, the results of STAPLE method were inferior to those of MV method. When the number of atlas match was increased, the performance of STAPLE method was improved: the generated contours were comparable to those generated with MV method. The results indicate that STAPLE method performs better when the number of atlas match is larger.

**FIGURE 6:**
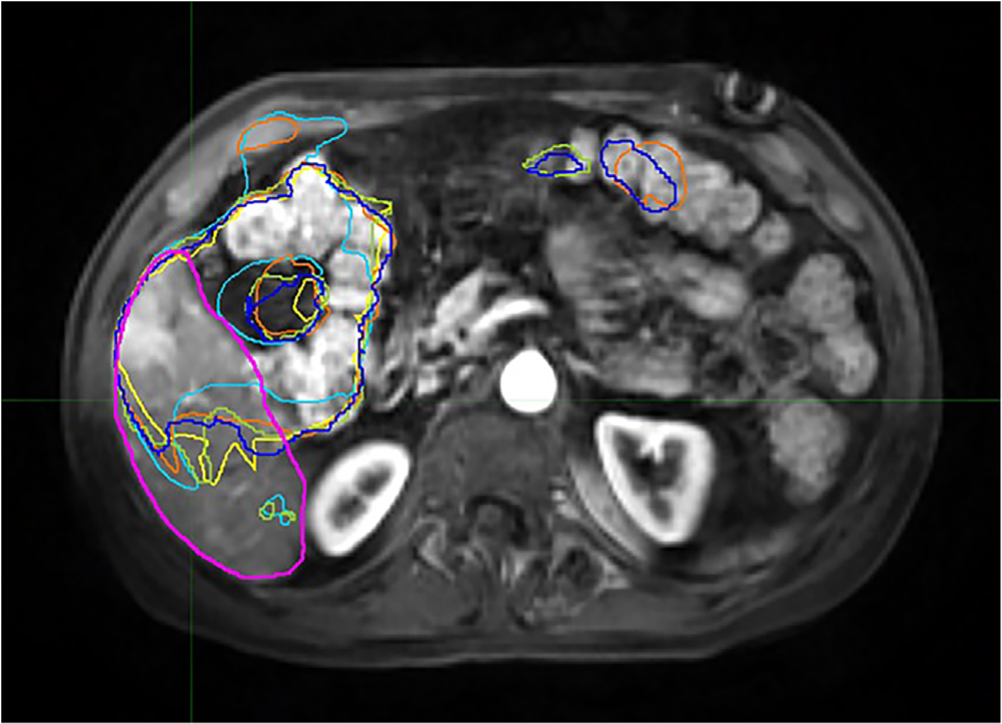
A case where high image intensities of nearby organs and heterogeneity of liver brought challenges to the auto‐segmentations: Norm (light blue), Norm_Match3_MV (red‐Orange), Norm_Match3_Staple (light green), Norm_Match5_MV (yellow), and Norm_Match5_Staple (dark blue). Manually‐delineated contour is in magenta.

With normalized deformable registration, the auto‐segmentations with 3‐atlas match using MV method generated better results than the auto‐segmentations with single‐atlas match. But there was no significant improvement in the auto‐segmentation when the number of matched atlases was increased from 3 to 5. It indicates a quick saturation in the improvement with the increase of matched atlases. The limited improvement in the auto‐segmentation might be due to the image quality. Image quality brings challenges in MR liver auto‐segmentations. Image noise and artifacts, etc., make it difficult to auto‐segment a liver. The patients included in the study were liver cancer patients. Heterogeneous image intensities of tumor and liver tissue brought challenges to the atlas‐based auto‐segmentation to generate accurate contours. Similar or higher image intensities of nearby tissues and organs also brought challenges to the auto‐segmentation. Figure 6 shows an example, where due to the high image intensities of nearby organs which had contrast agent, and the heterogeneity of the liver, the auto‐segmentation performances were poor. Further, variation of liver shape and size between a test case and an atlas subject brings challenges to the atlas‐based auto‐segmentation. The current study has the limitation: the atlas which included 20 subjects, was small. A larger atlas may provide better matches and improve the performance of the auto‐segmentation.

The results indicate that normalized deformable registration and multi‐atlas match, instead of the default setting, should be used in the auto‐segmentation. In the study, the contours generated in Norm_Match3_MV have an average DSC of 0.83, which is comparable to that in Yan et al.’s study^13^ where an atlas‐based method was developed to improve liver auto‐segmentation in MR images. To the authors’ knowledge, there have no publications studying the MIM atlas auto‐segmentation for liver delineation in MR images. Based on the study, it is recommended that for liver auto‐segmentation in MR images with Atlas Segment, one shall try applying the normalized deformable registration, which may generate better results if the images are contrast enhanced. Multi‐atlas match can be used to improve the auto‐segmentation. If the number of atlas match is small (e.g., < 5), MV method should be used. If the number of atlas is large, STAPLE method may be used. As STAPLE method takes into account of the quality of each contour, it may produce better results in the cases where the number of atlas match is large. Because the improvement brought by multi‐atlas match may be limited and the increase of matched atlases will increase the computation time, one may need to choose an appropriate number of matched atlases for the auto‐segmentation, to balance the limited improvement and the computation time.

The results show that average RA are 1.00‐1.01 and majority of RA are close to 1, that is, Y‐90 activities calculated using the auto‐segmented liver volumes are close to the accurate activities calculated using the manually‐delineated liver volumes, which indicates that the atlas‐based auto‐segmentation can be used to generate initial liver contours in MR images for Y‐90 SIRT procedures. The auto‐segmented liver contours, with physicians’ review and slight editing, will be able to use to generate accurate Y‐90 activities.

MIM software with AI‐based auto‐segmentation module became commercially available recently. It is not widely available in clinic yet. We anticipate testing AI‐based auto‐segmentations of liver in MR images for SIRT applications in the near future.

## 5 CONCLUSIONS

The settings used for the auto‐segmentation were recommended. With an appropriate setting, Atlas Segment can be applied to generate initial liver contours in MR images for resin Y‐90 SIRT, which can be used for activity calculations after physicians review.

## AUTHOR CONTRIBUTIONS

Jun Li designed the study, analyzed the data, and wrote the manuscript. Rani Anne contoured the volumes.

## CONFLICT OF INTEREST STATEMENT

The authors declare no conflicts of interest.

## Data Availability

The data that support the findings of this study are available on request.
